# Performance on the APACHE II, SAPS II, SOFA and the OHCA score of post-cardiac arrest patients treated with therapeutic hypothermia

**DOI:** 10.1371/journal.pone.0196197

**Published:** 2018-05-03

**Authors:** Jea Yeon Choi, Jae Ho Jang, Yong Su Lim, Jee Yong Jang, Gun Lee, Hyuk Jun Yang, Jin Seong Cho, Sung Youl Hyun

**Affiliations:** 1 Department of Emergency Medicine, Gachon University Gil Medical Center, Incheon, South Korea; 2 Department of Emergency Medicine, Mokpo Hankook Hospital, Mokpo, Jeollanam-do, South Korea; 3 Department of Thoracic and Cardiovascular Surgery, Gachon University Gil Medical Center, Incheon, South Korea; Azienda Ospedaliero Universitaria Careggi, ITALY

## Abstract

**Objective:**

This study assessed the ability of the Acute Physiologic and Chronic Health Evaluation (APACHE) II score, Simplified Acute Physiology Score (SAPS) II, Sequential Organ Failure Assessment (SOFA) score, and out-of-hospital cardiac arrest (OHCA) score to predict the outcome of OHCA patients who underwent therapeutic hypothermia (TH).

**Methods:**

This study included OHCA patients treated with TH between January 2010 and December 2013. The APACHE II score, SAPS II, and SOFA score were calculated at the time of admission and 24 h and 48 h after intensive care unit admission. The OHCA score was calculated at the time of admission. The area under the curve (AUC) of the receiver operating characteristic curve and logistic regression analysis were used to evaluate outcome predictability.

**Results:**

Data from a total of 173 patients were included in the analysis. The APACHE II score at 0 h and 48 h, SAPS II at 48 h, and OHCA score had moderate discrimination for mortality (AUC: 0.715, 0.750, 0.720, 0.740). For neurologic outcomes, the APACHE II score at 0 h and 48 h, SAPS II at 0 h and 48 h, and OHCA score showed moderate discrimination (AUC: 0.752, 0.738, 0.771, 0.771, 0.764). The APACHE II score, SAPS II and SOFA score at various time points, in addition to the OHCA score, were independent predictors of mortality and a poor neurologic outcome.

**Conclusions:**

The APACHE II score, SAPS II, SOFA score, and OHCA score have different capabilities in discriminating and estimating hospital mortality and neurologic outcomes. The OHCA score, APACHE II score and SAPS II at time zero and 48 h offer moderate predictive accuracy. Other scores at 0 h and 48 h, except for the SOFA score, are independently associated with 30-day mortality and poor cerebral performance.

## Introduction

Cardiac arrest (CA) is a major health problem. The global incidence of emergency medical services (EMS) treated adults for out-of-hospital cardiac arrest (OHCA) is 62 cases per 100,000 persons [1]. As post resuscitation care advanced, the rate of good neurologic outcome in witnessed ventricular tachycardia/ventricular fibrillation arrest was reported to be 59.7–66.5% [2,3]. However, the survival rates of all CA patients remain considerably low. Approximately two-thirds of initially resuscitated patients subsequently die in the hospital. A large number of these in-hospital deaths are due to post-cardiac arrest syndrome involving multiple organs [4]. Even if the patients survive past hospital discharge, many survivors have significant neurological sequela. In two randomized clinical trials, therapeutic hypothermia (TH) has been shown to improve neurological outcomes in adults who remained comatose after initial resuscitation from OHCA [5,6]. TH has been implemented as a standardized treatment for post-resuscitation care after CA [7].

The prediction of neurologic outcomes in comatose resuscitated patients is very important in order to reduce unnecessary costs, facilitate organ donation, and direct counseling with the patients’ families. The severity of illness scoring systems, such as the Acute Physiologic and Chronic Health Evaluation (APACHE) II score [8], Simplified Acute Physiology Score (SAPS) II [9], and Sequential Organ Failure Assessment (SOFA) score [10], were designed to predict in-hospital mortality or severity in critically ill patients. The purpose of these scores is to provide a value that can be averaged for a group of patients, not tell the individual patient's chance of survival. In several recent studies [11–14], these scores have been tested on their ability to predict the outcome of post-cardiac arrest patients, and they showed moderate predictive accuracy. Originally, these scores were calculated using the worst values from data collected in the first 24 hours (h) after intensive care unit (ICU) admission and were therefore not immediately available at the time of admission. For this reason, the APACHE III [15] score and SAPS III [16,17] calculated using data available at the time of admission were tested in recent studies [12,13,18]. However, these two scores showed lower predictive accuracy than the APACHE II score and SAPS II. The OHCA score was developed to predict the outcome at the time of ICU admission in resuscitated OHCA patients [19]. This score showed good calibration and high discrimination in predicting poor outcomes [20]. However, all of these scores have been tested without regard to the effects of TH implementation. TH modifies the prognostic accuracy of parameters for outcome prediction [21]. It is unclear whether the use of TH influences the utility of scoring systems for predicting outcome. It is worth evaluating the performance of these scoring systems in a homogeneous group of CA patients who have undergone TH.

The purpose of this investigation was to evaluate the performance of APACHE II score, SAPS II, SOFA score, and OHCA score for predicting the mortality and poor neurologic outcome of OHCA patients who underwent TH.

## Materials and methods

### Setting

This was a retrospective study conducted at an urban tertiary care academic medical center with 1300 inpatient beds and nearly 100,000 emergency department (ED) visits per year. The study was conducted in the ED and ICU from January 1, 2010 to December 31, 2013.

### Population

The study population included all adult (age > 18 years) patients who were admitted to the ICU after OHCA, with successful return of spontaneous resuscitation (ROSC) and underwent TH. CA patients with a primary traumatic etiology and in-hospital cardiac arrest (IHCA) patients were excluded.

### Post-resuscitation care

Post-resuscitation care was based on the recommendations and guidelines of the International Liaison Committee on Resuscitation (ILCOR) [4]. The decision on whether to initiate TH was made by the treating physician according to the TH inclusion criteria: 1) successful ROSC from CA; 2) comatose patient (Glasgow coma scale [GCS] of less than or equal to 8); 3) mean arterial pressure of greater than or equal to 60 mmHg with or without the use of vasopressor agents. If the patient did not satisfy the TH indications, TH was not performed. In addition, patients were excluded if the guardian refused treatment because of malignancy with multiple metastases or old age. All patients were given mechanical ventilation, sedatives, analgesics and muscle relaxants during TH. The target temperature of 33°C was induced by either an external or endovascular cooling device in addition to ice packs, 4°C normal saline infusion, and bladder irrigation. External cooling was performed using a water blanket (Blanketrol® II, Cincinnati Sub-Zero Products, OH, USA) or a pad (ArcticGelTM Pads & Arctic Sun® 2000, Medivance, CO, USA). Endovascular cooling was performed using a catheter (Cool Line® Catheter & CoolGard 3000®, ZOLL, MA, USA). After 24 hours of the maintenance phase, rewarming began with a rate of 0.2~0.3°C/h up to 36.0°C, and normothermia was maintained for 72 hours.

### Data collection and definitions

Data collected included patient demographic information, comorbid conditions, variables that are necessary for calculating severity scores, mortality and neurologic status at 30 days. CA event data was recorded in the Utstein style [22]. The APACHE II score and SAPS II were originally measured during the first 24 hours after ICU admission but in this study, the APACHE II score, SAPS II, and SOFA score were calculated at the time of admission (0 h), from the admission to the first 24 h (24 h), and from the 24 h to the 48 h (48 h) based on the worst vitals and laboratory findings in each 24-h interval. Initial physiologic values used at 0 h were not included in the calculation at 24 h. If there were any missing data points, they were calculated according to the methodology described by Vincent et al [23]. The GCS, which is included in calculation of the score, used the last value measured before sedation for TH [24]. The OHCA score was calculated based on the initial recorded rhythm, no-flow interval, low-flow interval, serum creatinine, and arterial lactate [19]. The neurologic outcome was measured using cerebral performance category (CPC) score [25]. The CPC score at 30 days after ICU admission was assessed by an ICU doctor via direct examination when the patient stayed in our hospital. The other patients were evaluated by a patient’s caregiver or doctor in the transferred hospital via telephone to define the CPC score at 30 days after CA. Patients were divided into two groups by CPC score. A CPC score of 1 or 2 is considered a good neurologic outcome, while a CPC score of 3, 4, or 5 is considered a poor neurologic outcome [5,6]. The study was approved by the Institutional Review Board at Gachon University Gil Medical Center (IRB No. GCIRB2015-100).

### Statistical analysis

Continuous variables were presented as the mean with standard deviation (SD) or the median with inter-quartile range (IQR) according to data distribution. Categorical variables were presented as frequency with percentage. The t-test was used to compare differences between groups when the dependent variable was continuous. A chi-square test was used to examine the association between categorical variables. The discrimination of these scores was assessed using area under the curve (AUC) of the receiver operator characteristic (ROC) curve. An AUC value of 0.9–1.0 indicates excellent, 0.8–0.89 good, 0.7–0.79 moderate, 0.6–0.69 poor and 0.5–0.59 not useful. Multivariate analysis was performed to identify the predictors for mortality and poor neurologic outcome at 30 days. Variables had a p-value of 0.2 or less on univariate analysis entered in the backward stepwise elimination. A p-value less than 0.05 was considered statistically significant. Statistical analysis was performed using SPSS version 18.0 (SPSS, Chicago, IL, USA) and MedCalc program version 12.7.7.0 (MedCalc Software, Mariakerke, Belgium).

## Results

**Fig 1:**
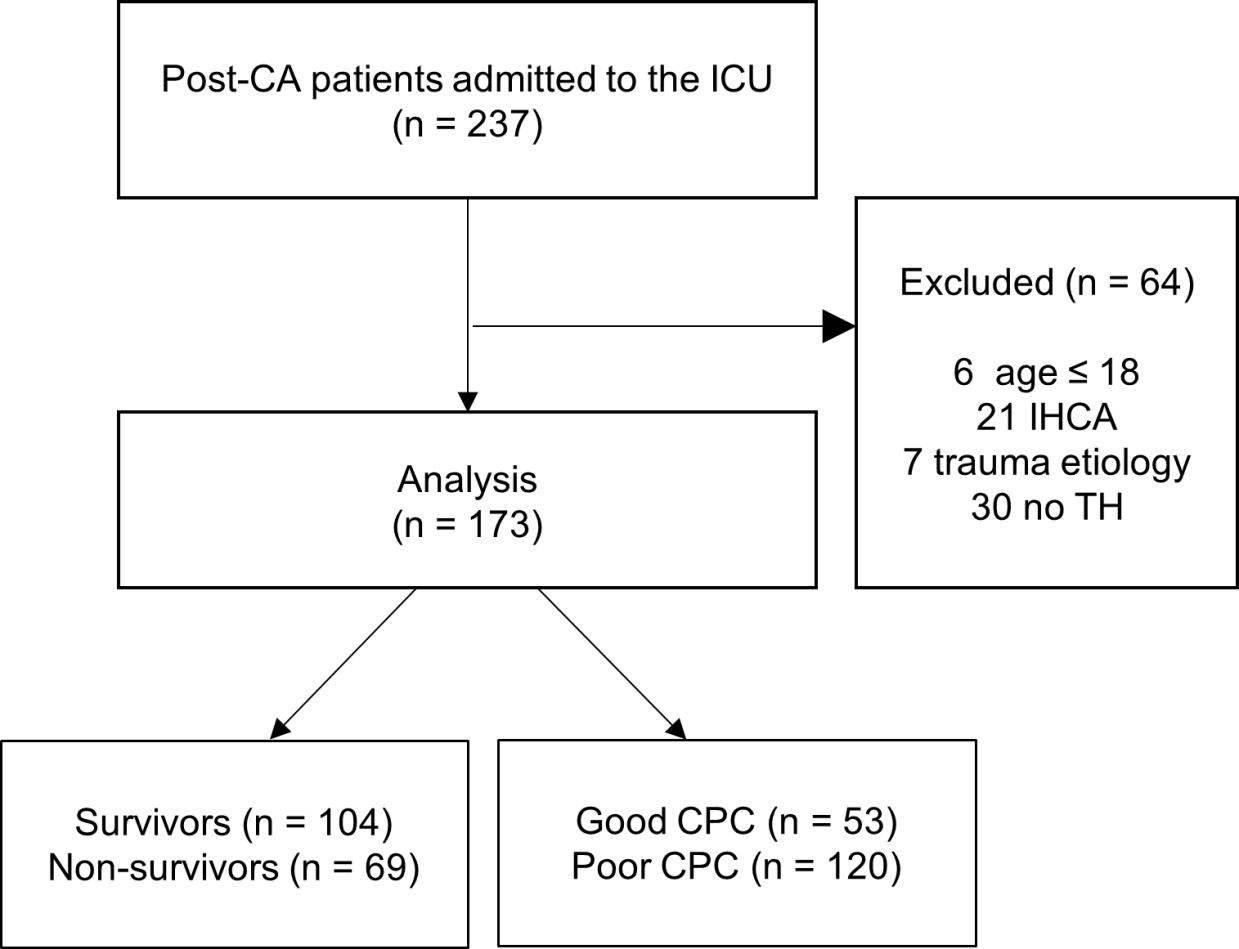
Flow diagram of post-CA patients between January 2010 and December 2013.

Of the 237 patients, 173 were included, and 64 patients with the following conditions were excluded (Fig 1, S1 File). The mean age was 53 years (SD: ±14.8), and 118 patients (68.2%) were male. Baseline characteristics and cardiac arrest data are summarized in Table 1. Hospital mortality within 30 days was 39.9%. Good neurologic outcome at 30 days after ICU admission was recorded in 53 (30.6%) patients. Internal cooling was performed in 74 of 173 patients and external cooling was performed in 99 patients. There was no difference between the survivors/non-survivors (p = 0.301) and good/poor CPC groups (p = 0.226). The median time between cardiac arrest to emergency department arrival was 27 minutes (IQR: 18–86). The median time between emergency department arrival to admission was 113 minutes (IQR: 73–176).

**Table 1:** Comparison of characteristics of survivors/non-survivors and good/poor CPC groups.

	All (n = 173)	Survivors (n = 104)	Non-survivors (n = 69)	p value	Good CPC (n = 53)	Poor CPC (n = 120)	p value
Age, year (±SD)	53 (14.8)	52 (13.6)	55 (16.4)	0.183	49 (13.3)	55 (15.2)	0.026
Male (%)	118 (68.2)	76 (73.1)	42 (60.9)	0.091	42 (79.2)	76 (63.3)	0.038
Comorbidities (%)							
Hypertension	56 (32.4)	36 (34.6)	20 (29.0)	0.438	18 (34.0)	38 (31.7)	0.766
Diabetes	32 (18.5)	26 (15.4)	16 (23.3)	0.196	6 (11.3)	26 (21.7)	0.106
Heart failure	19 (11.0)	13 (12.5)	6 (8.7)	0.433	5 (9.4)	14 (11.7)	0.665
CAD	31 (17.9)	24 (23.1)	7 (10.1)	0.030	12 (22.6)	19 (15.8)	0.282
CVD	9 (5.2)	5 (4.8)	4 (5.8)	1.000	1 (1.9)	8 (6.7)	0.279
COPD	12 (6.9)	4 (3.8)	8 (11.6)	0.067	2 (3.8)	10 (8.3)	0.348
CKD	8 (4.6)	5 (4.8)	3 (4.3)	1.000	1 (1.9)	7 (5.8)	0.438
Cause of CA (%)							
Cardiac	112 (64.7)	81 (77.9)	31 (44.9)	<0.001	51 (96.2)	61 (50.8)	<0.001
Non-cardiac	61 (35.3)	23 (22.1)	38 (55.1)		2 (3.8)	59 (49.2)	
Witnessed arrest (%)	133 (76.9)	88 (84.6)	45 (65.2)	0.003	47 (88.7)	86 (71.7)	0.014
Bystander CPR (%)	57 (32.9)	38 (36.5)	19 (27.5)	0.217	19 (35.8)	38 (31.7)	0.590
Initial shockable rhythm (%)	58 (33.5)	47 (45.2)	11 (15.9)	<0.001	35 (66.0)	23 (19.2)	<0.001
Interval from collapse to ROSC, min (IQR)	30 (23.5–42.0)	28 (21.0–40.0)	36 (27.0–46.0)	<0.001	25 (18.0–36.5)	31 (26.0–43.8)	0.001
Epinephrine before ROSC, mg (IQR)	2.0 (2.0–4.0)	2.5 (1.0–4.0)	2.0 (2.0–4.0)	0.295	2.0 (1.0–4.5)	2.0 (2.0–4.0)	0.202
Initial lactate, mmol/L (IQR)	7.9 (5.8–10.5)	7.2 (4.9–10.2)	9.3 (7.1–11.3)	0.001	6.8 (4.5–10.5)	8.7 (6.6–10.8)	0.008

SD: standard deviation, CPR: cardiopulmonary resuscitation, ROSC: return of spontaneous circulation, IQR: inter-quartile range, CA: cardiac arrest, CPC: cerebral performance category, CAD: coronary artery disease, CVD: cerebrovascular disease COPD: chronic obstructive pulmonary disease, CKD: chronic kidney disease

The serial mean values of each scoring system according to mortality and neurologic outcome are shown in Table 2. When each score was measured over time, there was a significant difference between the survivor and non-survivor groups and between the good CPC and poor CPC groups.

**Table 2:** APACHE II score, SAPS II, SOFA score, and OHCA score according to time the period with 30-day mortality and neurologic outcome.

		Mortality		
		Survivors (n = 104)	Non-survivors (n = 69)	p-value
APACHE II,	0-h	20.4 (5.4)	24.1 (4.5)	<0.001
mean (± SD)	24-h	26.6 (4.9)	29.5 (5.3)	<0.001
	48-h	23.2 (4.4)	26.8 (5.0)	<0.001
SAPS II	0-h	50.1 (10.5)	57.9 (11.2)	<0.001
	24-h	58.4 (11.6)	64.3 (11.8)	0.002
	48-h	52.9 (10.3)	62.0 (10.7)	<0.001
SOFA	0-h	8.1 (2.8)	9.5 (2.7)	0.001
	24-h	9.5 (2.6)	10.6 (2.9)	0.011
	48-h	9.1 (2.2)	10.4 (2.8)	0.003
OHCA	(±SD)	32.2 (13.7)	43.4 (13.3)	<0.001
		Neurologic outcome		
		Good CPC (n = 53)	Poor CPC (n = 120)	p-value
APACHE II,	0-h	18.6 (4.8)	23.3 (5.0)	<0.001
mean (± SD)	24-h	25.7 (4.6)	28.6 (5.2)	0.001
	48-h	21.9 (4.4)	25.8 (4.7)	<0.001
SAPS II	0-h	45.5 (10.0)	56.6 (10.3)	<0.001
	24-h	55.3 (11.3)	63.0 (11.6)	<0.001
	48-h	48.9 (10.5)	59.6 (10.0)	<0.001
SOFA	0-h	7.5 (2.9)	9.1 (2.7)	<0.001
	24-h	9.1 (2.6)	10.3 (2.7)	0.011
	48-h	8.7 (2.3)	9.9 (2.6)	0.006
OHCA		27.2 (14.4)	40.8 (12.6)	<0.001

APACHE: acute physiologic and chronic health evaluation, SAPS: simplified acute physiology score, SOFA: sequential organ failure assessment, OHCA: out-of-hospital cardiac arrest, SD: standard deviation, CPC: cerebral performance category.

Time-dependent AUC values for each scoring system to predict mortality and poor neurologic outcome at 30 days are shown in Table 3. The APACHE II score at 0-h (AUC: 0.715) and 48-h (AUC: 0.750), SAPS II at 48-h (AUC: 0.720), and OHCA score (AUC: 0.740) had a moderate ability to discriminate survivors from non-survivors. The APACHE II score at 0-h (AUC: 0.752) and 48-h (AUC: 0.738), SAPS II at 0-h (AUC: 0.771) and 48-h (AUC: 0.771), and OHCA score (AUC: 0.764) showed moderate discrimination for poor neurologic outcomes. The SOFA score showed poor discrimination of both mortality and neurologic outcomes. All scores measured at 24-h had poor discrimination. In addition, we analyzed whether mortality and neurologic outcome could be predicted by GCS alone. The initial GCS measured before sedation was not useful for discrimination for mortality (AUC: 0.486, 95% CI: 0.393–0.580, P = 0.773) and poor neurologic outcome (AUC: 0.518, 95% CI: 0.429–0.607, P = 0.688).

**Table 3:** AUC values for each scoring system to predict 30-day mortality and neurologic outcomes.

	AUC (95% CI)		
	0-h (n = 173)	24-h (n = 168)	48-h (n = 160)
**Mortality**			
APACHE II	0.715 (0.642–0.781)	0.657 (0.580–0.728)	0.750 (0.628–0.775)
SAPS II	0.686 (0.612–0.755)	0.640 (0.563–0.713)	0.720 (0.644–0.788)
SOFA	0.641 (0.564–0.712)	0.607 (0.529–0.682)	0.637 (0.558–0.712)
OHCA	0.740 (0.668–0.805)		
**Poor neurologic outcome**			
APACHE II	0.752 (0.681–0.814)	0.646 (0.568–0.781)	0.738 (0.663–0.804)
SAPS II	0.771 (0.701–0.831)	0.681 (0.605–0.750)	0.771 (0.698–0.834)
SOFA	0.669 (0.594–0.739)	0.619 (0.541–0.692)	0.625 (0.545–0.700)
OHCA	0.764 (0.693–0.826)		

AUC: area under the curve, CI: confidence interval, APACHE: acute physiologic and chronic health evaluation, SAPS: simplified acute physiology score, SOFA: sequential organ failure assessment, OHCA: out-of-hospital cardiac arrest.

**Fig 2:**
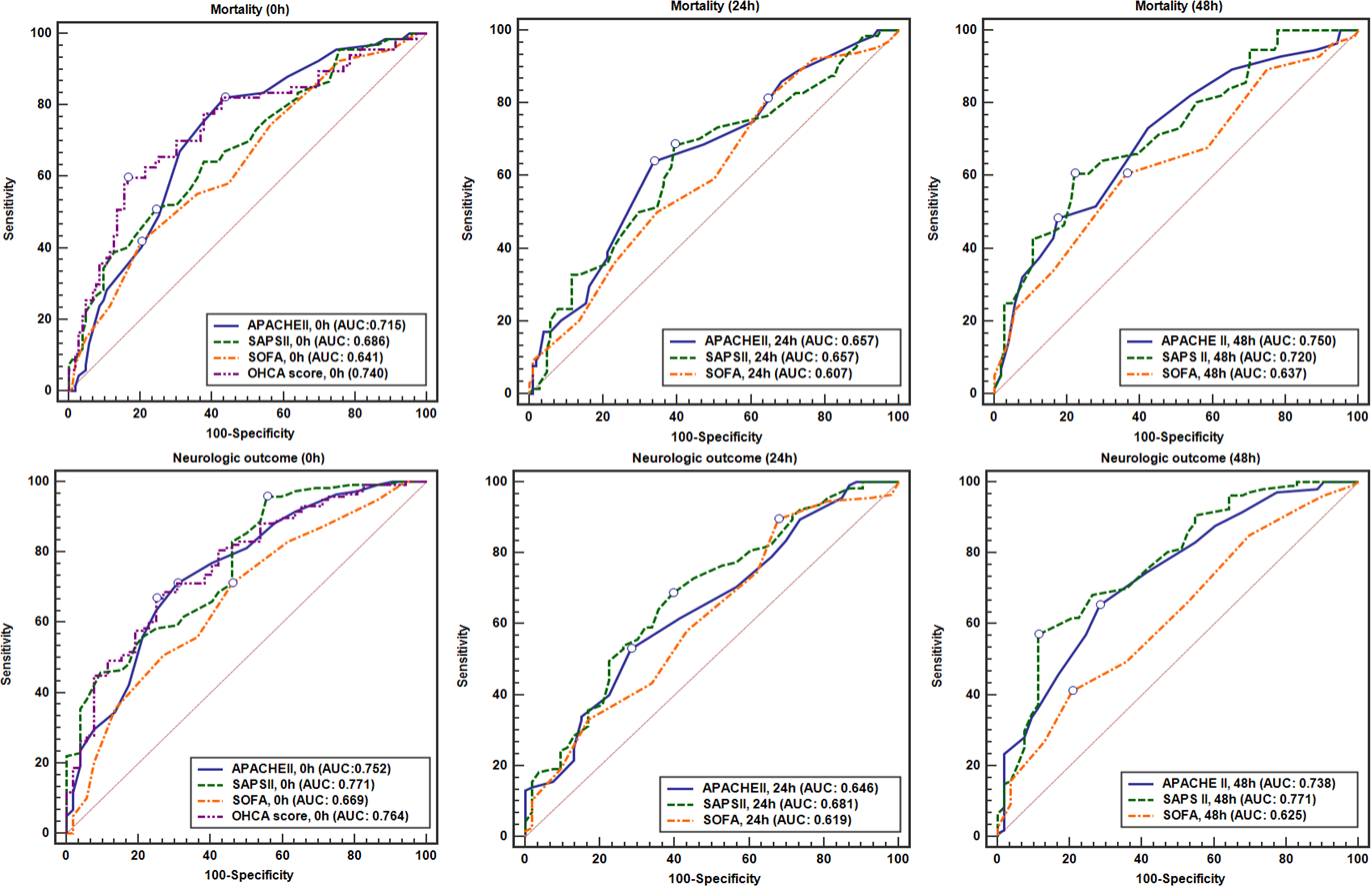
The ROC curves for severity scores and OHCA score over time.

The ROC curves for the APACHE II score, SAPS II, SOFA score, and OHCA score are shown in Fig 2. In predicting mortality, there was no statistical difference of the AUC values between scorings for each time period. In the neurologic outcome prediction, the AUC value of SAPS II measured at 0-h was statistically higher than the AUC value of SOFA score (P = 0.039). The AUC values for the SAPS II and APACHE II score measured at 48-h were statistically higher than the AUC value for the SOFA score (P = 0.001, 0.022).

Severity scores and OHCA score association at each time point are shown in Table 4. Multivariate logistic regression was performed for each severity scoring system and time interval with other variables. Variables included in the analysis were age, sex, witnessed arrest, comorbidities (diabetes, coronary artery disease, COPD), initial shockable rhythm, cause of cardiac arrest, interval from collapse to ROSC, and initial lactate. As a result, 10 models for mortality and 10 models for poor CPC were performed to predict significant predictors.

**Table 4:** Multivariate analysis of severity scoring systems with 30-day mortality and poor neurologic outcome.

		Mortality			
		Unadjusted OR (95% CI)	p-value	Adjusted OR (95% CI)	p-value
APACHE II	0-h	1.151(1.077–1.230)	<0.001	1.101(1.024–1.185)	0.009
	24-h	1.117(1.046–1.193)	0.001	1.078(0.991–1.173)	0.082
	48-h	1.173(1.087–1.267)	<0.001	1.162(1.052–1.284)	0.003
SAPS II	0-h	1.070(1.036–1.104)	<0.001	1.047(1.010–1.085)	0.012
	24-h	1.044(1.015–1.073)	0.003	1.047(1.013–1.083)	0.006
	48-h	1.090(1.049–1.132)	<0.001	1.107(1.056–1.160)	<0.001
SOFA	0-h	1.200(1.070–1.346)	0.002	1.147(1.004–1.311)	0.044
	24-h	1.165(1.033–1.315)	0.013	1.114(0.964–1.286)	0.144
	48-h	1.242(1.082–1.427)	0.002	1.294(1.064–1.573)	0.010
OHCA		1.067(1.038–1.097)	<0.001	1.057(1.027–1.088)	<0.001
		Poor neurologic outcome			
		Unadjusted OR (95% CI)	p-value	Adjusted OR (95% CI)	p-value
APACHE II	0-h	1.233(1.133–1.343)	<0.001	1.131(1.019–1.256)	<0.001
	24-h	1.135(1.052–1.225)	0.001	1.047(0.954–1.150)	0.332
	48-h	1.230(1.122–1.349)	<0.001	1.154(1.034–1.289)	0.011
SAPS II	0-h	1.121(1.075–1.169)	<0.001	1.086(1.033–1.142)	0.001
	24-h	1.064(1.030–1.099)	<0.001	1.042(0.998–1.087)	0.061
	48-h	1.121(1.072–1.171)	<0.001	1.086(1.033–1.141)	0.001
SOFA	0-h	1.242(1.095–1.408)	0.001	1.209(1.022–1.430)	0.027
	24-h	1.179(1.036–1.342)	0.013	1.102(0.927–1.310)	0.271
	48-h	1.217(1.054–1.405)	0.007	1.103(0.914–1.333)	0.307
OHCA		1.077(1.046–1.109)	<0.001	1.061(1.029–1.094)	<0.001

OR: odd ratio, CI: confidence interval, APACHE: acute physiologic and chronic health evaluation, SAPS: simplified acute physiology score, SOFA: sequential organ failure assessment, OHCA: out-of-hospital cardiac arrest.

Variables included in analysis were age, sex, witnessed arrest, comorbidities (diabetes, coronary artery disease, COPD), initial shockable rhythm, cause of cardiac arrest, interval from collapse to ROSC, and initial lactate.

In the logistic regression analysis, the APACHE II score (0-h OR: 1.101; 95% CI: 1.024–1.185; 48-h OR: 1.162, 95% CI: 1.052–1.284) and SAPS II (0-h OR: 1.047, 95% CI: 1.010–1.085; 48-h OR: 1.107, 95% CI: 1.056–1.160) were determined to be independent factors in predicting mortality. The APACHE II score (0-h OR: 1.131, 95% CI: 1.019–1.256; 48-h OR: 1.154, 95% CI: 1.034–1.289) and SAPS II (0-h OR: 1.086, 95% CI: 1.033–1.142; 48-h OR: 1.107; 95% CI: 1.052–1.164) were also independently associated with poor neurologic outcomes. The OHCA score was a significant predictor of death and poor CPC.

## Discussion

To the best of our knowledge, this is the first study to simultaneously evaluate the performance of different scoring systems, such as the APACHE II score, SAPS II, SOFA score, and OHCA score, in a homogeneous group of OHCA patients who underwent TH. In this investigation, we found that the APACHE II score, SAPS II, and OHCA score had a moderate ability to discriminate outcomes following CA. When examining various severity scores by time, we were able to identify that the scores at certain time points were independently associated with mortality and poor neurologic outcome. It should be emphasized, however, that the scoring systems are intended to predict disease severity and mortality, not to determine patient’s medical management. In clinical settings, this result should not influence the decision to discontinue treatments.

The SAPS II at 48-h and the APACHE II score at 0-h and 48-h time points and the OHCA score were moderate predictors of 30-day mortality. The SAPS II and APACHE II score at 0-h and 48-h time points and the OHCA score were moderate predictors of the 30-day neurologic outcome. The SAPS II and APACHE II score at 24-h and the SOFA score at all time-points were poor predictors. The largest AUC was obtained by the SAPS II at 0 h (AUC: 0.771) and 48 h (AUC: 0.771) for neurologic outcome and the APACHE II score at 48 h (AUC: 0.750) for mortality. In multivariate logistic regression analysis, the SAPS II and APACHE II score at the 0-h and 48-h time points were associated with 30-day mortality and poor neurologic outcome. We also analyzed whether any of the variables except the severity scores were related to mortality or poor CPC. Variables such as cause of CA (non-cardiac origin), interval form collapse to ROSC, initial non-shockable rhythm, and initial lactate level were significant predictors of both mortality and poor neurologic outcome. However, these factors are not included in the calculation of the APACHE II score, SAPS II, and SOFA score. As noted in previous studies [11–14], severity scoring systems should be considered to not be specifically developed for CA patients’ evaluation. Pre- and intra-arrest conditions and factors contribute substantially to the severity of the post-cardiac arrest syndrome and on outcomes [26]. If the severity scores, such as the APACHE II score and SAPS II, are increasingly combined with related factors, they may improve predictability of outcome in OHCA patients.

In a study by Donnino et al. [11], the APACHE II score measured at 0 h (AUC: 0.58) was not useful to predict neurologic outcome in OHCA patients. The performance score increased incrementally over the next 24-h (AUC: 0.74), 48-h (AUC: 0.79), and 72-h (AUC: 0.90) time points. However, in our series, the APACHE II score at 0 h (AUC: 0.752) showed moderate discrimination. These differences are explained by the following reasons. First, the GCS score included in the calculation of severity scoring systems in our study was the last measured GCS before sedation for TH. In the study by Livingstan et al. [24], using the GCS recorded before sedation increased the discrimination of APACHE II score. Second, other studies include a heterogeneous group with TH implementation. On the other hand, our study only included patients undergoing TH that may have more effect on results. The differences in the patient groups included in the analysis, the outcome predictability of scoring systems could differ.

The OHCA score incorporates variables available early after ROSC, such as no-flow and low-flow intervals, serum lactate and creatinine levels at admission, and initial cardiac rhythm. Previous validation studies of OHCA score showed a similar good performance in terms of discrimination, which supports the generalizability of the score despite the differences in age, no-flow time, initial rhythm, and the rate of TH. In our study, OHCA score achieved moderate discrimination (AUC: 0.740 for mortality, 0.764 for neurologic outcome) for the 30-day outcome, but it was lower compared with that of previous studies [18–20]. In addition to the OHCA score, the Pittsburgh Cardiac Arrest Category (PCAC) was showed AUC of 0.79–0.82 for mortality and functional outcome [27]. This can be due to differences in patient baseline characteristics, emergency medical system, and TH application rate. Specifically, previous studies with high discriminatory performance have only a small percentage (11% and 34%) of TH application compared with 100% in our study. This suggests that TH affects the performance of the OHCA score.

The time-dependent predictive performances of the APACHE II score and SAPS II were moderate at 0 h and 48 h, but the predictability of all scoring systems declined at 24 h. Regarding adjusted OR, the scores at 24 h were not predictors of outcome except for SAPS II at 24 h for mortality. This tendency seems to be due to the effect of TH. Induction of TH usually starts at the time of admission. Parameters needed to calculate scores at 24 h were obtained during the first 24 hours after ICU admission. Parameters obtained in this period may reflect the effect of TH because the target temperature is maintained for 24 hours after induction of TH. TH affects various clinical and laboratory parameters [28]. Mean arterial pressure increases slightly (±10mmHg), and cardiac output decreases due to a decrease in heart rate. In blood samples from hypothermic patients, PO2 and PCO2 are overestimated, while pH is underestimated, and thus metabolic acidosis looks more severe. White blood cells and platelets decrease and hematocrit mildly increases. These physiologic changes generally do not require treatment and also do not affect prognosis. However, the severity scores were changed because these parameters are used to calculate severity scores. It is thought that outcome predictability is reduced when the score is calculated using measured parameters during 24 hour hypothermic treatment.

In our study, survival was 104/173 patients but good CPC occurred in only 53. This is because of many patients with CPC 4 are in the survival group compared with previous studies. The end-of-life decision such as the withdrawal of life-sustaining therapy (WLST) is a very sensitive and difficult problem due to medical, ethical, and economical reasons. In Korea, WLST is not permitted unless the patient is brain death and donate organ. Therefore, comatose resuscitated patients receive intensive care support for prolonged periods compared with Europe and may lead to different outcome of early stage. As a result, discrimination for mortality decreases compared with that for poor neurologic outcome.

This study has several limitations because it was a single-center study and because a sample size calculation was not performed. In addition, the severity scoring was performed within the first 48 hours after ICU admission. As discussed, the effect of hypothermia including rewarming is not resolved within this time. In the case of the APACHE score and SAPS, the temperature variable is included in calculations. In our study, no correction was made for the temperature effect, thus the results may look worse. The TH also affects other variables such as vital signs and laboratory tests’ results, especially the 24-h outcome. The clearance of drugs such as sedatives and muscle relaxants may not have been metabolized within 48 hours due to the influence of TH. If these score calculations were extended to 72 hours after ICU admission, facilitation of the identification of changes in scores over time and clearer determination of the effect of TH would be possible. Finally, although this study is meaningful to analyze only patients who underwent TH, patients who were too unstable initially to be treated with TH were excluded, thus possibly affecting the outcome due to bias.

## Conclusions

The OHCA score and most severity scores of different time points, except scores measured at 24 h, are significantly associated with mortality and poor neurologic outcome. The OHCA score, 0-h and 48-h scores of the APACHE II score and SAPS II offer moderate prediction accuracy ability in OHCA patients who underwent TH; however, the SOFA score remained poor. To improve the outcome predictability of CA patients, development of a new scoring system including cardiac arrest-related variables is needed.

## Supporting information

S1 File Data collection of 173 including patients. General characteristics, data for calculating severity scores at 0, 24, 48-hour and the outcome are shown in the each sheet.
(XLSX)
